# Effects of preoperative sagittal spinal imbalance on pain after lateral lumbar interbody fusion

**DOI:** 10.1038/s41598-022-06389-z

**Published:** 2022-02-22

**Authors:** Akihiko Hiyama, Hiroyuki Katoh, Daisuke Sakai, Masato Sato, Masahiko Watanabe

**Affiliations:** Department of Orthopaedic Surgery, Tokai University School of Medicine, Shimokasuya, Isehara, Kanagawa 259-1193 Japan

**Keywords:** Neurology, Neurological disorders, Quality of life, Neurosurgery

## Abstract

Sagittal misalignment has been associated with negative quality of life (QOL). However, there is no report on whether differences in preoperative sagittal misalignment in patients with lumbar degenerative diseases affect postoperative results after lateral lumbar interbody fusion (LLIF). We investigated whether preoperative sagittal alignment influences the correction of alignment after surgery and whether the preoperative sagittal alignment affects the rating of low back pain, leg pain, and leg numbness. The subjects were 81 patients (48 male, 33 females, average age at surgery 70.2 years) who underwent anterior–posterior combined surgery with LLIF and percutaneous pedicle screws from May 2018 to July 2020. Cluster analysis was performed using the preoperative sagittal vertical axis (SVA) value, and patients were classified into two groups (group 1; n = 30, SVA = 129.0 ± 53.4 mm, group 2; n = 51, SVA = 30.8 ± 23.5 mm). Baseline demographics and treatment data were compared between groups. Sagittal and pelvic parameters and pain scores, such as low back pain, leg pain, and leg numbness, were also compared. Operative time, blood loss, and length of hospital stay did not differ significantly between groups. The changes (Δ) in SVA and lumbar lordosis (LL) for all patients from before to after surgery were not significant (ΔSVA; p = 0.218, ΔLL; p = 0.189, respectively). The SVA, LL, and PI − LL changed significantly after the surgery in group 1, but no marked improvement in sagittal imbalance was obtained after LLIF surgery. The improvement in each pain score from before to after the surgery did not differ significantly between groups. LLIF surgery has a limited chance of recovering sagittal imbalance. However, postoperative low back pain, leg pain, and leg numbness may be improved by LLIF surgery, regardless of the preoperative sagittal alignment.

## Introduction

Sagittal misalignment as an adult spinal deformity (ASD) has attracted attention because of its association with negative quality of life (QOL) and increased disability^1–3^. An increased sagittal vertical axis (SVA) and pelvic incidence − lumbar lordosis (PI − LL) mismatch are strongly related to adverse patient-reported outcomes^3,4^. Thus, the goals of surgical correction involve optimizing the PI − LL and SVA to achieve global sagittal balance. Various procedures have been reported for planning the corrective surgery for treating ASD^5–7^. One widely accepted radiological target for achieving spinopelvic harmony via corrective surgery for ASD is to keep the pelvic incidence (PI)–lumbar lordosis (LL) within 10°^4,8,9^. Because the SVA more sensitively represents sagittal alignment and correlates strongly with the PI − LL mismatch, it is also used for planning surgical correction in ASD patients. Sagittal malalignment was defined as a sagittal vertical axis (SVA) ≥ 50 mm according to the Scoliosis Research Society-Schwab classification^9^.

Since its introduction in 2006^10^, lateral lumbar interbody fusion (LLIF) surgery has been used to treat various spinal pathologies^11,12^. Some groups have adopted it as an adjunct in corrective surgery for spinal deformity^13–15^. There is a wealth of data to show that LLIF surgery is useful for obtaining indirect decompression of the spine for treating lumbar degenerative diseases (LDDs)^16–20^.

Factors that can predict the success of indirect decompression with LLIF, including patient and surgical factors, particularly the cage position, have been investigated but are still debated^18,21–24^. In general, it makes sense to place the LLIF cage anterior to obtain the degree of LL^23^, but cases have been reported in which indirect decompression may not be accepted^18^. In addition, although some spinal parameter correction rates after LLIF surgery have been reported, there remains limited evidence about the effectiveness of LLIF surgery to correct sagittal deformities^25–28^. Similarly, there are few reports of whether and how preoperative sagittal balance affects pain in specific areas, such as low back pain (LBP), leg pain (LP), and leg numbness (LN), after LLIF surgery.

Therefore, the purpose of our study was to evaluate whether preoperative sagittal alignment influences the correction of alignment after LLIF surgery and whether the preoperative sagittal alignment affects the rating of LBP, LP, and LN.

## Results

### Patient demographics

During the study period, a total of 120 patients received LLIF in our institutions, and 81 patients (48 male and 33 females) with an average of 70.2 ± 10.4 years were evaluated. Patients whose data were incomplete or could not be followed up were excluded. The demographic and operative characteristics are detailed in Table 1. LLIF was performed at 110 levels, and 58 (71.6%) patients underwent a single-level procedure. The most commonly treated level was L4/5 (59/110, 53.6%). When the sagittal imbalance was defined as SVA ≥ 50 mm, 40 patients (42/81, 51.9%) were classified as having a sagittal imbalance.

**Table 1:** Demographic information.

Characteristic	Values
Total number	81
Age (years), mean (SD)	70.2(10.4)
≧ 65 years, n (%)	65 (80.2)
**Sex, n (%)**	
Male	48 (59.3)
Female	33 (40.7)
Height (cm), mean (SD)	159.0 (9.7)
Body weight (kg), mean (SD)	61.7 (12.2)
BMI (kg/m^2^), mean (SD)	24.3 (3.6)
Number of levels treated, n (mean)	110 (1.4)
**Number of levels treated, n (%)**	
1 level	58 (71.6)
2 levels	17 (21.0)
3 levels	6 (7.4)
**Operated level, n**	
L1/2	2
L2/3	11
L3/4	38
L4/5	59
Average OR time (min), mean (SD)	109.2 (37.0)
Average Blood loss (ml), mean (SD)	85.9 (118.8)
CRP on POD1, mean (SD)	3.3 (2.5)
Average length of stay (days), mean (SD)	15.1 (4.2)

*SD* standard deviation.

### Comparison of clinical outcomes and radiological assessment

Of the 81 patients, 30 were in group 1 with a high SVA (16 men, 14 females, average age 71.1 years), and 51 were in group 2 (32 men, 19 females, average age 69.7 years). A power analysis performed to detect the difference and showed 0.929 (effect size d = 0.8, alpha = 0.05, total sample size = 81, two-tailed). Age, sex distribution, height, body weight, and BMI did not differ significantly between the two groups. The number of levels treated, operative time, blood loss, and length of stay did not differ between the two groups. However, the C-reactive protein level on the day after surgery was lower in group 2. The rates of thigh pain and motor weakness, postoperative complications peculiar to LLIF surgery did not differ significantly between the two groups. Comparison of spinal parameters showed that preoperative LL differed significantly between groups 1 and 2 (24.3 ± 16.8° vs. 40.5 ± 12.3°, respectively) (Table 2). Given that the average PT in the elevated SVA group (group 1) is not high, most patients bend forward to relieve their stenosis.

**Table 2:** Comparison of demographic and treatment data between two groups.

Characteristic	Group 1	Group 2	p-value^‡^
SVA (mm), mean (SD)	129.0 (53.4)	30.8 (23.5)	
No. of patients	30	51	
Age (years), mean (SD)	71.1 (9.9)	69.7 (10.8)	0.670
**Sex, n (%)**			
Male	16	32	0.408
Female	14	19	
Height (cm), mean (SD)	157.7(9.6)	159.7 (9.7)	0.363
Body weight (kg), mean (SD)	60.8 (13.2)	62.3 (11.6)	0.588
BMI (kg/m^2^), mean (SD)	24.2 (3.2)	24.4 (3.9)	0.876
Number of levels treated, n (mean)	46 (1.5)	64 (1.3)	0.122
Average OR time (min)	118.4 (42.1)	103.8 (33.0)	0.153
Average Blood loss (ml)	121.8 (167.3)	64.8 (71.5)	0.079
CRP on POD1, mean (SD)	4.5 (3.2)	2.5 (1.6)	< 0.001*
Average Length of stay (days)	15.8 (4.5)	14.7 (3.9)	0.279
No. of motor weakness (%)	6 (20.0)	8 (15.7)	0.762
No. of thigh pain (%)	7 (23.3)	9 (17.6)	0.572
**Preoperative parameter**			
CR Cobb (°)	10.7 (8.7)	6.3 (5.5)	0.049*
LL (°)	24.3 (16.8)	40.5 (12.3)	< 0.001*
TK (°)	21.8 (11.8)	22.8 (10.3)	0.679
PI (°)	50.4 (7.4)	50.5 (8.8)	0.969
PT (°)	23.2 (7.8)	21.5 (7.2)	0.325
SS (°)	27.2 (8.3)	29.0 (8.7)	0.374

*SD* standard deviation.

*Statistically significant.

^‡^Comparison between two groups.

Table 3 summarizes the pre-and postoperative sagittal parameters. In group 1, ΔSVA (− 34.0 ± 65.3 mm, p = 0.008), ΔLL (6.5 ± 14.0°, p = 0.021), and ΔPI − LL (− 4.9 ± 12.8°, p = 0.045) were significant from preoperative to postoperative. In group 2, ΔSVA (9.3 ± 25.1 mm, p = 0.011) was significant from preoperative to postoperative. ΔLL was significantly larger (1.6 ± 10.9°, p = 0.011), and ΔPI − LL was significantly improved (0.9 ± 7.3°, p = 0.029) in group 1 with high SVA compared with group 2. The pelvic parameters ΔPI, ΔPT, and ΔSS did not differ significantly between the two groups.

**Table 3:** Preoperative, postoperative, and change from pre- to postoperative sagittal measurements.

	Preoperative	Postoperative	ΔPost–pre	p-value^†^
**SVA (mm)**				
Group 1	129.0 (53.4)	95.0 (48.8)	− 34.0 (65.3)	0.008*
Group 2	30.8 (23.5)	40.1 (34.9)	9.3 (25.1)	0.011*
ALL	67.2 (60.5)	60.4 (48.3)	− 6.7 (48.8)	0.218
p value^‡^	< 0.001*	< 0.001*	0.001*	
**LL (°)**				
Group 1	24.3 (16.8)	30.7 (15.7)	6.5 (14.0)	0.021*
Group 2	40.5 (12.3)	39.5 (11.6)	− 1.0 (8.0)	0.363
ALL	34.7 (15.7)	36.3 (13.9)	1.6 (10.9)	0.189
p value^‡^	< 0.001*	0.020*	0.011*	
**TK (°)**				
Group 1	21.8 (11.8)	21.4 (12.8)	− 0.6 (5.6)	0.723
Group 2	22.8 (10.3)	24.4 (9.1)	1.5 (5.6)	0.057
ALL	22.4 (10.8)	23.3 (10.6)	0.8 (5.6)	0.183
p value^‡^	0.679	0.278	0.108	
**PI (°)**				
Group 1	50.4 (7.4)	52.0 (8.1)	1.6 (4.3)	0.058
Group 2	50.5 (8.8)	50.4 (7.7)	− 0.1 (5.9)	0.872
ALL	50.5 (8.3)	51.0 (7.8)	0.5 (5.4)	0.412
p value^‡^	0.969	0.371	0.173	
**PT (°)**				
Group 1	23.2 (7.8)	23.1 (7.5)	− 0.2 (5.5)	0.867
Group 2	21.5 (7.2)	21.3 (6.9)	− 0.2 (5.5)	0.777
ALL	22.2 (7.4)	22.0 (7.1)	− 0.2 (5.5)	0.742
p value^‡^	0.325	0.289	0.969	
**SS (°)**				
Group 1	27.2 (8.3)	28.9 (9.2)	1.7 (7.3)	0.203
Group 2	29.0 (8.8)	29.1 (7.7)	0.1 (5.7)	0.914
ALL	28.3 (8.6)	29.0 (8.2)	0.7 (6.3)	0.325
p value^‡^	0.374	0.948	0.261	
**PI − LL (°)**				
Group 1	26.2 (14.9)	21.3 (13.8)	− 4.9 (12.8)	0.045*
Group 2	10.0 (9.6)	10.9 (9.9)	0.9 (7.3)	0.388
ALL	16.0 (14.1)	14.7 (12.5)	− 1.3 (10.0)	0.265
p value^‡^	< 0.001*	< 0.01*	0.029*	

^†^Comparison with pre op.

^‡^Comparison between two groups.

*Statistically significant.

**Figure 1:**
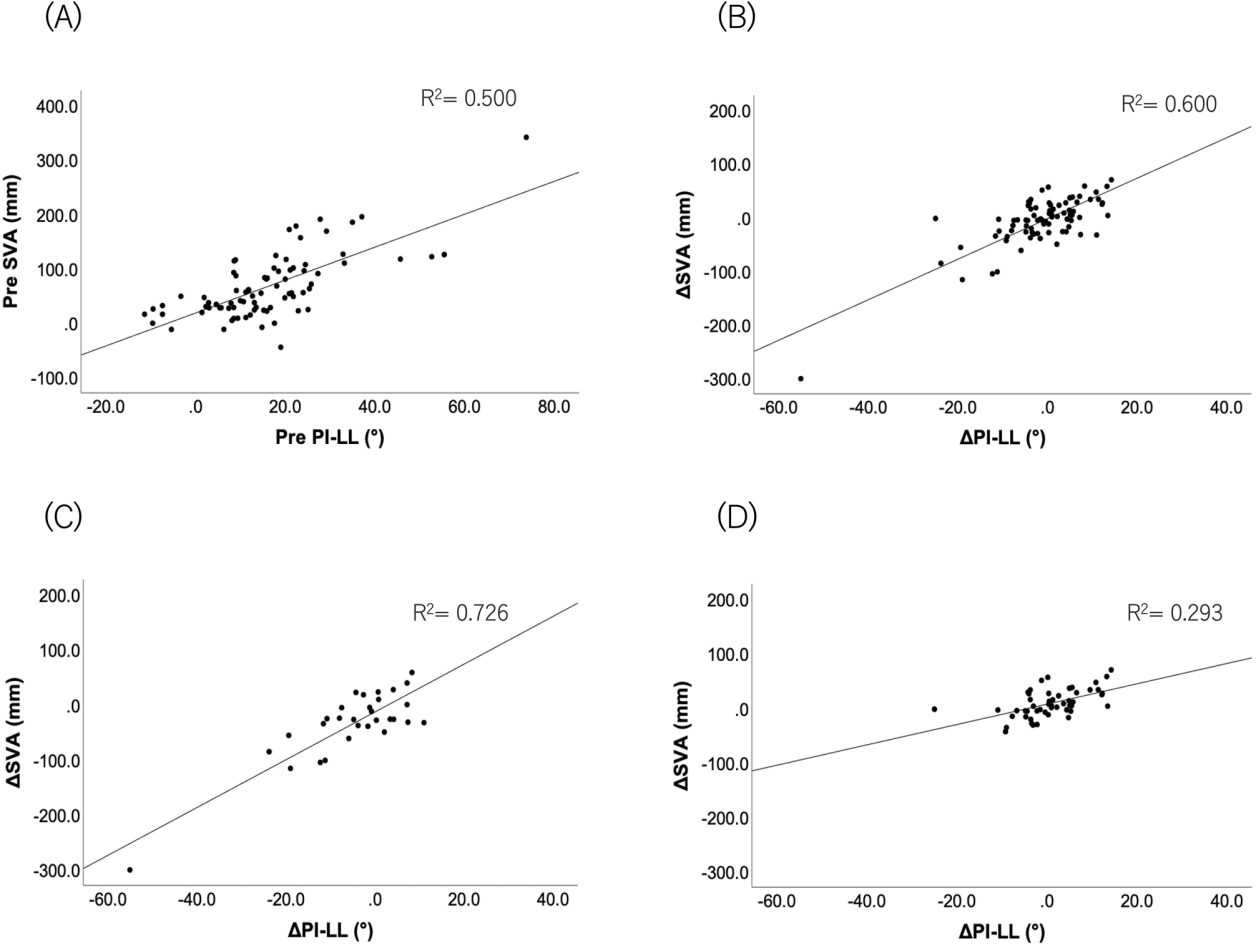
Correlations between SVA (*y*-axis) and PI − LL (*x*-axis). Each plot represents 81 cases. (**A**) Preoperative and (**B**) Δ (postoperative–preoperative) correlations. Each plot represents the case of (**C**) group 1 (n = 30) and (**D**) group 2 (n = 51). Δ (postoperative–preoperative) correlations. *SVA* sagittal vertical axis, *PI − LL* pelvic incidence minus lumbar lordosis.

We found strong correlations between the preoperative SVA and PI − LL in all 81 patients (r = 0.647, p < 0.001) and between the changes in these parameters (r = 0.584, p < 0.001). The correlation coefficients between ΔSVA and ΔPI − LL were also statistically significant 0.607 (p < 0.001) and 0.562 (p < 0.001) in groups 1 and 2, respectively (Fig. 1).

To assess the degree of indirect decompression resulting from LLIF surgery, Magnetic Resonance Imaging (MRI) scan was used to compare the CSA of the dural sac preoperatively and immediately after the operation. In all 81 patients, the average CSA increased from 60.2 ± 34.0 to 84.2 ± 36.4 mm^2^ from before to after the operation. The preoperative CSA (63.0 ± 34.2 vs 58.2 ± 35.5 mm^2^, p = 0.448), postoperative CSA (86.0 ± 37.7 vs 83.0 ± 36.7 mm^2^, p = 0.686), and ΔCSA (23.0 ± 18.8 vs 24.8 ± 24.8 mm^2^, p = 0.986) did not differ between groups (data not shown).

### Comparison of pain scores

Numeric rating scale (NRS) scores were obtained for LBP (NRS_LBP_), LP (NRS_LP_), and LN (NRS_LN_). Preoperatively, all patients had NRS scores indicating LBP (mean NRS_LBP_ 6.3 ± 2.6), LP (mean NRS_LP_ 6.8 ± 2.8), or LN (mean NRS_LN_ 6.3 ± 3.2), but these scores did not differ significantly between the two groups. The NRS_LBP_ scores one year after surgery were 4.0 ± 3.4 and 2.4 ± 2.8 for groups 1 and 2, respectively (p = 0.054), and the NRS_LP_ scores one year after surgery were 2.6 ± 2.7 and 1.8 ± 2.3 for groups 1 and 2, respectively (p = 0.150). Postoperative NRS_LN_ score were statistically significant (3.4 ± 2.8 and 2.1 ± 2.8, p = 0.019, respectively). In both groups, postoperative pain improved one year after the operation, but the improvements in each NRS score did not differ significantly between the two groups (Table 4).

**Table 4:** Preoperative, postoperative, and change from pre- to postoperative each NRS scores in the two groups.

	Preoperative	Postoperative	ΔPost–pre	p-value^†^
**NRS**_**LBP**_				
Group 1	6.5 (2.7)	4.0 (3.4)	− 2.6 (3.4)	< 0.001*
Group 2	6.2 (2.7)	2.4 (2.8)	− 3.8 (3.9)	< 0.001*
All	6.3 (2.6)	3.0 (3.0)	− 3.4 (3.8)	< 0.001*
p value^‡^	0.474	0.054	0.180	
**NRS**_**LP**_				
Group 1	6.6 (2.4)	2.6 (2.7)	− 4.3 (2.7)	< 0.001*
Group 2	6.9 (3.0)	1.8 (2.3)	− 5.1 (3.7)	< 0.001*
All	6.8 (2.8)	2.0 (2.4)	− 4.8 (3.4)	< 0.001*
p value^‡^	0.280	0.150	0.078	
**NRS**_**LN**_				
Group 1	6.5 (2.9)	3.4 (2.8)	− 3.4 (3.4)	< 0.001*
Group 2	6.1 (3.3)	2.1 (2.8)	− 4.0 (4.0)	< 0.001*
All	6.3 (3.2)	2.5 (2.8)	− 3.8 (3.8)	< 0.001*
p value^‡^	0.701	0.019*	0.281	

*NRS* numeric rating scale, *NRS*_*LBP*_ NRS for low back pain, *NRS*_*LP*_ NRS for leg pain, *NRS*_*LN*_ NRS for leg numbness.

^†^Comparison with pre op.

^‡^Comparison between two groups.

*Statistically significant.

A power analysis performed to detect the correlation (effect size d = 0.5, alpha = 0.05, two-tailed) showed 0.999, 0.874, 0.979 for total sample sizes 81, 30, and 51, respectively. The correlations between ΔSVA and NRS for LBP, LP, and LN were not significant in either group (Table 5).

**Table 5:** Spearman correlations mean (Spearman’s *r*) between ⊿SVA and each pain score.

	∆SVA	NRS_LBP_	NRS_LP_	NRS_LN_
**All (n = 81)**				
ΔSVA	1.000			
ΔNRS_LBP_	− 0.014	1.000		
ΔNRS_LP_	− 0.061	0.569***	1.000	
ΔNRS_LN_	− 0.194	0.487***	0.633***	1.000
**Group 1 (n = 30)**				
ΔSVA	1.000			
ΔNRS_LBP_	0.116	1.000		
ΔNRS_LP_	0.132	0.546**	1.000	
ΔNRS_LN_	0.021	0.364*	0.531**	1.000
**Group 2 (n = 51)**				
ΔSVA	1.000			
ΔNRS_LBP_	0.010	1.000		
ΔNRS_LP_	− 0.010	0.607***	1.000	
ΔNRS_LN_	− 0.187	0.507***	0.673***	1.000

*SVA* sagittal vertical axis, *NRS* numeric rating scale, NRS scores for low back pain (NRS_LBP_), for leg pain (NRS_LP_), and for leg numbness (NRS_LN_).

*p < 0.05, **< 0.01, ***< 0.001 indicates significant differences.

## Discussion

This is the first study to evaluate whether the sagittal balance in patients with LDD affects the improvement in pain one year after LLIF surgery. At present, various approaches are used in the ever-evolving context of spinal surgery^29^. LLIF surgery is now accepted as an effective treatment option for patients with LDD. Retrospective studies of indirect decompression surgery have investigated sagittal alignment changes after LLIF surgery^30,31^. Some groups have suggested that LLIF can increase segmental lordosis (SL) more than does transforaminal lumbar interbody fusion^32,33^. Acosta et al. reported that SL significantly increased by 2.9°, despite no significant changes in LL and SVA after the LLIF surgery^30^. Another study reported an increase in SL of 2.4°–2.7° after LLIF surgery^17^. A larger interbody cage is placed during LLIF than in surgery using the posterior approach, which results in more significant endplate contact for LLIF. Therefore, patients should benefit from a healthy biomechanical environment for fusion and segmental deformity correction. Another study reports that single position LLIF surgery can reduce operation time as minimally invasive spinal treatments^17^.

Understanding the limitations of LLIF is as important as knowing its strengths. It was previously reported that patients with claudication had sagittal imbalance, a higher SVA value, lower LL, and greater pelvic retroversion^34^. Sagittal imbalance is associated with poor QOL and is a source of LBP^2^. In addition, increasing the mechanical load on the lumbar spine because of PI − LL inconsistency, including SVA, raises concerns about adjacent segment disease^35^. For these reasons, improving spinal alignment is essential. Fuji et al. reported that sagittal imbalance returned to normal after decompression surgery in 43% of patients. They found that the prognostic factors for postoperative sagittal imbalance were a preoperative SVA value of > 69 mm and a PI − LL difference of > 11.5^36^. Madkouri et al. also reported that sagittal spinal imbalance improved after decompression surgery, suggesting that the preoperative partially forward-leaning posture is reversible and relieves pain. However, they also reported that patients presenting with an SVA value of > 100 mm showed residual imbalance^37^. These data suggest that the preoperative SVA value is associated with improving the SVA after surgery.

The present study found no significant changes in the LL of the 81 patients one year after LLIF surgery and that the overall sagittal alignment as shown by the SVA parameter or pelvic parameter did not change after LLIF surgery. The anterior sagittal imbalance is thought to reflect the loss of LL primarily, although it has been suggested that increasing the postoperative LL can improve the SVA in patients with a large preoperative SVA. In our study, Group 1 had an increased degree of LL after LLIF surgery. However, the mean postoperative SVA was 95.0 ± 48.8 mm in group 1, which fit the criterion for imbalance as an SVA of ≥ 50 mm. We believe that the potential for improving the sagittal imbalance by LLIF surgery is limited in patients with severe preoperative sagittal imbalance.

A systematic review of LLIF surgery reported significantly improved clinical outcomes in patients with LDD^11^. It was recently reported that LLIF could improve LBP, LP, and numbness in the lower extremities^38^. The present study examined whether the changes in NRS scores for LBP, LP, and LN after LLIF surgery were related to the preoperative SVA. We found no significant differences and that all NRS scores showed similar improvement, as previously reported^11,21^.

Our study has some limitations, such as the retrospective study, small number of patients, and the short follow-up period. Despite the significant results with this small population, a larger sample size of this cohort is needed to improve the statistical power. In addition, the LLIF surgery performed by spine surgeons is not always unified. Finally, the effects of preoperative comorbidities and the patient's social background should also be considered. Future prospective studies are needed to stratify the population to reduce possible confounding effects.

## Conclusions

LLIF surgery has a limited chance of recovering SVA in patients with preoperative sagittal imbalance. However, this study showed that indirect decompression using LLIF surgery might improve postoperative LBP, LP, and LN, regardless of the preoperative sagittal alignment.

## Material and methods

The study protocol was reviewed and approved by the Institutional Review Board of Tokai University School of Medicine, the House Clinical Study Committee, and the Profit Reciprocity Committee, and all of the methods were carried out in accordance with the ethical principles set out in the 1964 Declaration of Helsinki. This retrospective study was approved by the Institutional Review Board of Tokai University School of Medicine, and the requirement to obtain informed consent was waived (IRB approval no.: 21R-147). After institutional review board approval, a retrospective review of the clinical data from a single academic institution was performed. Patients were treated from May 2018 to July 2020.

### Included patients

The inclusion criteria included patients who underwent LLIF surgery for LDDs, including spondylolisthesis and spinal stenosis with instability. It is difficult to distinguish whether elevated SVA was from a spinal deformity or patients stooping forward due to spinal stenosis. Thus, we evaluated ASD or LDDs based on physical findings or pelvic parameters. Patients with LBP and mainly intermittent claudication and neurological symptoms, such as numbness, pain, and weakness in the lower extremities, were diagnosed with lumbar spinal stenosis.

Patients with significant lumbar scoliosis, grade 2 spondylolisthesis, or lumbar fracture were excluded. We also excluded patients who did not have adequate pre-and postoperative standing radiographs one year after surgery and those who could not evaluate their pain using a scoring system.

The preoperative information for all patients was assessed using standard radiographs, MRI scans, and computed tomography scans. The spine surgeon recorded the location of stenosis based on an evaluation of the preoperative imaging studies. The patient underwent indirect decompression with LLIF and posterior percutaneous pedicle screw fixation on the same day, and patients who underwent direct decompression were excluded. The operative approach for LLIF surgery has been detailed previously^17,39^.

**Figure 2:**
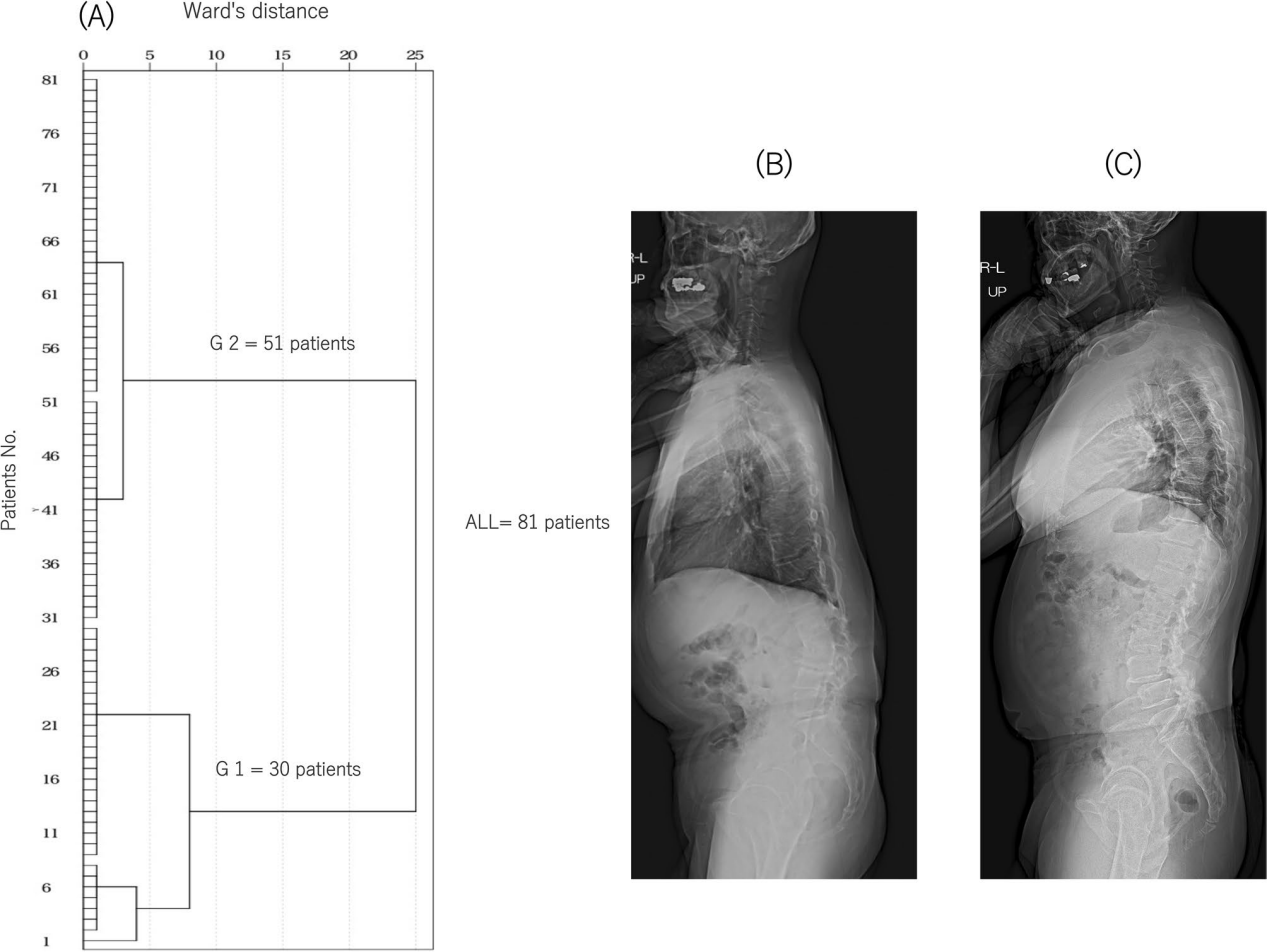
Dendrograms of the hierarchical classification of patients who received LLIF surgery (n = 81). (**A**) The numbers of patients in each cluster at different Ward’s distances are shown. The patients were classified into groups 1 (n = 30) and 2 (n = 51) from the cluster analysis. Standing full-length X-ray lateral views of typical cases in groups 1 (**B**) and 2 (**C**).

Cluster analysis was performed using the hierarchical cluster analysis procedure using IBM SPSS Statistics (version 23.0; IBM Corp., Armonk, NY, USA). Cluster analysis was used to classify the patients into two groups based on the preoperative SVA (Fig. 2): group 1 with a high SVA (129.0 ± 53.4 mm) and group 2 with an SVA close to normal (30.8 ± 23.5 mm).

### Surgical technique

The basic procedure of our LLIF was performed according to the surgical technique described by Ozgur et al.^10^. The technique has been explained in our previous papers, but we will briefly describe^17,39–41^. The surgery is performed for indirect decompression, and the emphasis is not on alignment correction. All patients underwent LLIF through a single incision, mini-open direct visualizing approach. Patients were placed in true lateral positions, and a horizontal skin incision was made. A blunt incision was made until it reached the vertebral body. The cartilage endplate was removed using a Cobb elevator and curette when treating the endplate. Cage size trials were followed by additional disc curettage and rasping of the endplates. The surgeon determined the appropriate cage size by combining preoperative images and intraoperative cage template findings. All LLIF segments were applied with supplemental percutaneous pedicle screw fixation.

### Radiological assessment

Standing full-length radiographs were evaluated preoperatively and one year after the surgery. Using X-rays of the whole spine with the patient in the standing position and standard measurements reported elsewhere^42^, we assessed the coronal Cobb angle, SVA, LL at T12-S1, thoracic kyphosis (TK) at T5-12, PI, pelvic tilt (PT), sacral slope (SS), and PI − LL. PI was measured as the angle between a line drawn perpendicular to the sacral endplate at its midpoint and a line drawn from the midpoint of the sacral endplate to the midpoint of the femoral head axis. LL was measured as the sagittal Cobb angle measured between the superior end plate of T12 and the superior endplate of S1. MRI was performed preoperatively and immediately after surgery to determine the cross-sectional area (CSA) of the spinal canal using the axial plane of T2-weighted images.

### Clinical assessment

The clinical records were reviewed retrospectively by identifying demographic data, including age, sex, height, body weight, and body mass index (BMI). The operative time, blood loss volume, level of LLIF surgery, length of hospital stay, and LLIF-specific complications (motor weakness and thigh pain) were quantified using surgical items and hospitalization records. Anterior thigh pain that occurred between the time of surgery and discharge was recorded as “thigh pain”. If there were clinical problems with hip flexion after LLIF, “motor weakness” was recorded. Motor weakness was evaluated using the Barthel Index (BI) as reported previously^16^. Briefly, the stair climbing score of the BI evaluates whether a person can climb and descend stairs safely using a 3-point scale (0 = unable; 5 = needs help, such as verbal, physical, or carrying aids; 10 = independent). Patients whose stair climbing score was lowered by one rank after surgery was considered a motor weakness.

The pain intensity was assessed using an NRS, and NRS scores were obtained for LBP (NRS_LBP_), LP (NRS_LP_), and LN (NRS_LN_) preoperatively and 1 year after the surgery. An 11-point scale was used in which 0 = no pain to 10 = worst pain or pain as bad as it could be.

### Statistical analysis

Statistical analyses were performed using IBM SPSS Statistics. All values are expressed as mean ± standard deviation. The Shapiro–Wilk test was used to confirm the normality of the data distribution. For the direct comparison of the two groups, Student's t-test was used to analyze normally distributed data, and the Mann–Whitney U test was used to analyze nonnormally distributed data. The Pearson or Spearman coefficient was used to identify significant correlations between pre-and postoperative spinal parameters and between the changes (Δ) in SVA and pain in each area. We used the G-Power Analysis software program to determine sample size validity (G*Power 3.1). Post-hoc analysis using G*Power 3.1 was performed to detect the correlation of subjects and the difference between two independent groups.

The type 1 error was set at 5% for all statistical analyses, and p < 0.05 was considered to be significant.
